# ‘Fostering transformative learning, self-reflexivity and medical citizenship through guided tours of disadvantaged neighborhoods’

**DOI:** 10.1080/10872981.2018.1537431

**Published:** 2018-11-01

**Authors:** E. Marshall Brooks, Mary Lee Magee, Mark Ryan

**Affiliations:** Department of Family Medicine and Population Health, Virginia Commonwealth University, Richmond, VA, USA

**Keywords:** Medical education, community tours, medical citizenship, transformative learning, underserved populations

## Abstract

**Background and objectives**: Medical school curricula increasingly seek to promote medical students’ commitment to redressing health disparities, but traditional pedagogical approaches have fallen short of this goal. The objective of this work was to assess the value of using community-based guided tours of disadvantaged neighborhoods to fill this gap.

**Methods:** A total of 50 second-year medical students participated in a guided tour of disadvantaged public housing neighborhoods in Richmond, Virginia. Students completed self-reflexive writing exercises during a post-tour debriefing session. Student writings were analyzed to assess the tour’s effect on their awareness of poverty’s impact on vulnerable populations’ health and wellbeing, and their personal reactions to the tour.

**Results:** Student writings indicated that the activity fostered transformative learning experiences around the issue of poverty and its effects on health and stimulated a personal commitment to working with underserved populations. Themes from qualitative analysis included: increased awareness of the extent of poverty, enhanced self-reflexive attitude towards personal feelings, biases and misperceptions concerning the poor, increased intentional awareness of the effects of poverty on patient health and well-being, and, encouragement to pursue careers of medical service.

**Conclusions:** This pilot demonstrated that incorporating self-reflexive learning exercises into a brief community-based guided tour can enhance the social consciousness of medical students by deepening understandings of health disparities and promoting transformative learning experiences.

## Introduction

The Institute of Medicine (IOM) suggests medical school curricula train students who are technically competent clinicians as well as engaged ‘medical citizens.’ [1,2] While modern medical training capably teaches students medical knowledge, it less effectively promotes a sense of medical citizenship—that is, a commitment to critically understand the systemic obstacles faced by, and proactively advocate for, local communities and their socioeconomic needs [3–6]. Although rarely integrated into formal medical school curricula, the idea of medical citizenship is not new. Writing in the late 1800s, German physician Rudolf Virchow famously asserted that ‘physicians are the natural advocates of the poor’ and ‘if medicine is really to accomplish its great task, it must intervene in political and social life’. Despite such calls, rapid advancements in the biomedical sciences and cultural shifts in the medical profession have rigidified medical curricula’s focus on the basic sciences and clinical skill building without equivalent attention to training in medical citizenship.

Recent efforts to integrate social determinants of health (SDOH) education into medical curricula have likewise insufficiently fostered a sense of medical citizenship. Although laudably seeking to increase students’ awareness of healthcare challenges in low income communities [7,8], health disparities education based on decontextualized statistical data and clinical rotations that expose students to safety-net healthcare settings often fail to promote ‘transformative learning experiences’—that is, deep seated, lasting changes to students’ personal perspectives and professional sensibilities[9]. What is more, such educational programs risk reifying negative stereotypes of low-income communities when the underlying socio-structural factors contributing to health disparities, and learners’ personal preconceptions of impoverished populations, are left uninterrogated [10–13]. Instead, transformative learning theory suggests that learners’ firmly ingrained cultural biases must first be disrupted before meaningful change can take hold. Only when educational activities promote ‘introspection, intersubjective reflection and collaboration, and a critical analysis of social conditions and injustice’[14] can learners develop a holistic and empathetic appreciation for how poverty impacts health and develop a personal commitment to social justice.

To achieve the sorts of transformative learning experiences necessary to promote medical citizenship, we developed and pilot tested a community-based guided tour of a public housing neighborhood with second year medical students as part of a four-year track preceptorship program focused on medically underserved populations. Unlike similar so-called ‘windshield tours’ and other SDOH experiential-learning activities [15–19], our community tour sought to not only educate students on SDOH and health disparities, but to foster meaningful shifts in personal commitments and professional sensibilities. To accomplish this, we incorporated self-reflexive exercises throughout the activity that cultivated students’ intentional awareness of structures of inequality, holistic understanding of how such structural inequalities impact health, and critical reflection about how personal, often emotion-laden, reactions to poverty shape one’s professional goals and sensibilities. Because medical students often come from contexts of privilege with incomplete or inaccurate preconceptions of low-income communities[20], integrating such activities into the second year of medical school is especially important to mitigate students’ preexisting biases and misconceptions before they interact with patients from local impoverished communities during clinical rotations.

In this brief report we describe this novel approach to conducting community-based, guided tours, and present preliminary results from a pilot study evaluating the feasibility and effectiveness of the tour for promoting transformative learning, self-reflexivity and a sense of medical citizenship among medical students. Through qualitative analysis of a self-reflexive writing exercise that students completed during the tour, we investigated how participation in the tour facilitated several key features of transformative learning and medical citizenship, including: awareness of social inequalities, knowledge of how poverty impacts health, a self-reflexive attitude towards personal feelings and biases, and motivation to pursue careers of medical service.

## Methods

In Fall 2015 and 2016, two cohorts of 25 (50 total) second-year medical students from the Virginia Commonwealth University (VCU) School of Medicine participated in an hour-long guided community bus tour of disadvantaged neighborhoods in Richmond, Virginia, immediately followed by a debriefing session and reflexive writing exercise. The tour was organized and led by staff members of a local, nonprofit organization with a 25-year history of serving the community. Students were members of a four-year track preceptorship program for medical students who have declared a commitment to working with medically underserved populations.

## Guided community bus tour

During an initial orientation session, staff members from the local, nonprofit organization presented the history and mission of the organization to introduce students to an effective example of community engagement and to prepare them for the community tour. The toured neighborhoods contained a concentration of public housing units and unaccredited public schools. While guiding the bus tour, staff members discussed the neighborhood’s social, economic and political history, the residents’ socioeconomic disadvantages, and descriptions of how this legacy of inequality impacts residents’ health and wellbeing. This included the impact of gentrification over the past several decades, growing disparities in home values between gentrified and non-gentrified neighborhoods, the dilapidated condition of section eight housing, prevalence and impact of violent crime, lack of amenities such as grocery stores and banks, and how the proximity of neighborhoods to a nearby prison, juvenile detention center and landfill shaped residents’ sense of place and personhood.

## Index-card emotion exercise

Prior to boarding the bus, students were provided small index cards and instructed to record any emotions or thoughts they spontaneously experienced while on the tour, as well as notes about what they were seeing or hearing when experiencing those thoughts or emotions. Students were instructed not to write their names on the cards, and informed that their responses would be used to facilitate a post-tour debriefing session (described below). This exercise sought to foster students’ intentional awareness of the social and structural inequalities impacting residents’ health and wellbeing, and to promote self-reflexive scrutiny of one’s emotions and reactions when encountering underserved communities and populations.

## Post-tour debriefing session

After the community tour students engaged in an hour long debriefing co-facilitated by two faculty members from the VCU Department of Family Medicine and Population Health, including one physician and one licensed clinical social worker, and staff from the local non-profit organization. VCU faculty members initiated the debriefing with a broad, open-ended question—‘What were your initial reactions during the tour?’ Students then took turns giving brief descriptions of their emotional reactions and observations. Periodically throughout the debriefing, VCU faculty members read aloud several of the index cards upon which students had recorded their emotional responses during the tour (see Table 1). After each reading the faculty facilitator asked the group to reflect on how their own experiences reflected or diverged from what was read, and asked follow-up, probing questions about the personal meaning, source, or underlying significance of whatever emotion or observation was being commented upon. Through such self-reflexive, iterative dialogue, this exercise sought to foster greater collective awareness of shared emotional experiences, and to promote a mindset of critical contemplation about one’s own experiences, reactions, and emotions.

**Table 1.** Examples of students’ self-reported emotions and thoughts during the tour.

Emotions			Thoughts and observations	
Negative	Defeated	Overwhelmed	‘Boundaries and blatant injustices faced by residents.’	‘While the rest of the city is improving, these communities have been left behind.’
	Frustrated	Shocked		
	Shame	Humbled	‘Seeing the prison system in direct line of sight between the community and downtown.’	‘I’m not different from these folks, but I’ve had so many more opportunities.’
	Guilt	Trapped		
	Disappointed	Surprised	‘How do you even make a dent in this sort of poverty?’	‘We haven’t passed a grocery store! How do people eat?’
	Lonely	Disgust		
	Dismayed	Hopeless	‘We place and concentrate such poverty to let it fester and cause worse conditions.’	‘The people/kids are set up for failure.’
	Sad	Heartbreak		
Positive	Inspired	Encouraged	‘I can see where I can be used to help uplift individuals in these communities.’	‘Inspired by development center dedication and persistence.’
	Hopeful	Connected		
	Curious	Empowered	‘Hope for future changes.’	‘Feeling of potential.’
	Excited	Motivated		

## Self-reflexive writing exercise

At the end of the debriefing session students were asked to write short responses to three open-ended questions: 1) What did you learn today? 2) What did you learn about yourself today? And, 3) What will you do differently as a result of your experience today? Having the students respond to the questions through an individual writing assignment, as opposed to in a group discussion, allowed for further critical self-reflexivity about personal biases, misconceptions and areas needing further growth without fear of judgement from peers or facilitators.

## Analysis of student writings

Student responses from the self-reflexive writing exercise were subjected to a qualitative content analysis using template and emergent coding processes [21,22]. Codes were derived from the literature on medical citizenship and transformative learning, which included themes related to critical understanding and intentional awareness of the extent and effects of poverty; self-reflexive attitude towards personal feelings, biases and misperceptions; intent to advocate or pursue social justice for underserved communities, and challenges and barriers to transformative learning and adopting qualities of medical citizenship. All three authors initially coded responses separately, and then met to review themes and discuss findings through an iterative process[22]. Analysis of the index cards with self-reported emotions consisted of sorting responses into ‘positive’ and ‘negative’ for descriptive purposes. All student writings were kept anonymous. The Institutional Review Board at VCU approved this study.

## Results

Students’ self-reported emotional reactions—positive and negative—and associated thoughts and observations during the tour are presented in Table 1. Results from students’ self-reflexive writing exercises are presented in Table 2. These are organized into themes spanning learning outcomes, personal impact of the community tour and potential challenges within such educational activities. Our findings indicate that the activity fostered four key dimensions of transformative learning and medical citizenship, including increases in: 1) awareness of the extent of poverty in surrounding communities; 2) self-reflexive attitude towards personal feelings, biases and misperceptions concerning the poor; 3) intentional awareness of how poverty shapes health and well-being; and, 4) motivation to pursue careers of medical service. Additionally, student responses revealed how this educational activity may be undermined by several potential challenges and barriers to transformative learning and developing a sense of medical citizenship. Example quotes are provided to illustrate students’ articulation of these themes.

**Table 2.** Themes and learning outcomes from the community tour as evidenced in students’ self-reflexive writings.

Themes	Learning outcomes	Sample of student responses
Awareness of poverty	Increased awareness of the extent and impact of poverty in local communities Recognition of how insulated from poverty medical students typically are	‘I am amazed at the despair and brokenness located just a few miles from where we live and study, completely unaware of the hardships going on blocks away.’ ‘It amazes me how close these densely disadvantaged communities can be to me without ever interacting with them.’
Critical understanding of how poverty impacts health and wellbeing	Fostered intentional awareness of patients’ perspectives, experiences, and lives at home and in their communities Increased professional sensitivity to how socio-economic barriers undermine patients’ ability to access, engage, and benefit from healthcare services	‘Reminded again about how complex and difficult it is to improve upon health and poverty.’ ‘I will choose to be more intentionally aware of who my future patients are as individuals and what challenges they face in order to successfully adopt healthy practices. Even what I consider to be the simplest plan or cheapest plan may seem difficult or be insurmountable to those with far less resources and opportunities.’ ‘I learned to never make assumptions about someone’s living conditions, for I may never truly know their home situation or difficulty in simply accessing food, medicine, healthcare, or an education.’
Self-reflexive attitude	Revealed latent feelings and attitudes towards underserved populations Exposed areas of intellectual and experiential ignorance regarding structural inequalities Critically interrogated the goals, benefits, and impact of the healthcare system vis a vis underserved communities Identified areas for further personal and professional growth	‘I realized how much sadness and compassion I have for people with unfortunate circumstances and how separate that is from pity.’ ‘I learned how little I know about poverty and the reality of what it looks like.’ ‘I may not be as culturally competent as I once thought. I do not understand a lot of the power dynamics or cultural dynamics at play.’ ‘We are probably benefiting more than the people we treat.’ ‘I only understand things at surface level and am constantly surprised and frustrated by its complexity and stagnation.’
Motivation to serve	Spurred proactive stance towards rectifying social injustices Renewed commitment to pursuing careers of medical service Inspired critical engagement with social, political and economic issues outside the immediate purview of clinical medicine	‘It is not enough to observe the injustice, but we must do what we can to take action to start impacting the people around us.’ ‘It reminded me why I am here. I’m grateful for the opportunity to learn about these broken communities so that I can go out when I am a physician and impact the community as a whole in which I serve.’ ‘I have frequently been plagued by self-doubt throughout medical school, and my belief in my ability to help others has declined secondary to that. But today, there were many moments I thought to myself, “I can do that!”’ ‘I felt an inclination to challenge and question much more strongly than I have before.’ ‘I think it underscores the fact that as people of power we have to consider the impact of our desires in policy on all parts of the city, not only for ourselves. We can’t let ourselves be the reason why neighborhoods like this can’t get better.’
Educational challenges related to transformative learning and medical citizenship	Developing personal ethos of medical citizenship potentially requires extensive experiential education on/in low-income communities Increased understanding of structural inequalities can be demoralizing if not paired with education on how to effect pragmatic solutions Inability to personally identify with underserved populations risks undermining transformative learning experiences Ambivalence towards the healthcare system as both the problem and solution to health care inequalities challenges students’ commitment to pursue careers of medical service	‘I don’t always see through the eyes of those less fortunate as much as I would like to think that I do. It’s hard to empathize or even have the energy or context of understanding.’ ‘I am not poor, black or even from the eastern states. That puts me so far from being able to help this community.’ ‘I often walk away from these sessions deeply upset because we are often talking about limitations and all the reasons that we may not be able to affect change.’ ‘A sense of “I’m not sure what to do next” sort of hit me time and time again.’ ‘It is hard to decide whether contributing in ways I can to broken systems is helping or hurting in the end.’

## Discussion

Students appeared to gain an appreciation for the complexity and difficulty of combating poverty, as well as realizing how little they understood about the causes and consequences of social inequality, a high-priority issue in an era in which many practicing physicians do not believe that health disparities exist[23]. Rather than discuss these issues as abstract concepts or statistics as might happen in a classroom setting, the visceral sights, sounds and smells of the community tour, combined with the post-tour self-reflexive discussion and writing exercises, seemed to allow students to experience, feel, and contemplate social and structural inequality in an ‘experientially near’ learning experience[24]. Further, reflecting expert advice to focus on provider attitudes and stereotyping in medical education [10,11,25], combining guided tours, reflexive writing and group discussion effectively spurred students to critically interrogate personal biases, more fully evaluate how structures of inequality shape a community’s health, and contemplate pursuing careers of medical service.

The tour’s strategy of exposing students to local communities affected by health disparities, a teaching methodology supported in the literature [26,27], also benefited students’ sense of preparation for future clinical work in community settings. Responding to expert suggestion that medical students develop the skills and intuition necessary to elicit information about patients’ social and cultural contexts, the fact that this community tour was located in neighborhoods in which many of the academic health center’s patients live provided some students with preparation and greater insight into how to care for patients they would soon be encountering during clinical rotations. This included considering how limited financial and social resources impacts patients’ ability to access healthcare and adhere to treatment plans, and avoiding making assumptions about patients’ motivations, desires, or abilities.

Despite potential educational benefits, community tours of impoverished neighborhoods present other surreptitious educational and ethical challenges. Like similar community-based education programs that work with disadvantaged populations, unmediated exposure to social inequality risks re-affirming negative stereotypes of poverty [10,12]. Participating in guided tours of low-income neighborhoods to which no direct aid or assistance will be offered in the context of the educational experience also risks crossing the line into poverty or slum tourism[28]. Particularly if educators are not mindful that the ostensibly benign act of touring potentially exoticizes community members and unintentionally fosters uncritical depictions of people’s differences. However, this can be mediated by properly preparing and debriefing students before and after the activity, or partnering with a known and respected community organization whose representatives can share community-based insights, provide counterpoints to dominant stereotypes, and highlight positive characteristics and resources in the community[29].

This study has several limitations. First, students with a latent propensity for working with underserved populations may have self-selected into the preceptorship program. Second, as this was a cross-sectional assessment of student responses, long-term fluctuations in empathy or actual career trajectories were not tracked. We are currently developing additional longitudinal and comparative studies to assess such outcomes across cohorts and between curricula at different schools of medicine.

This pilot, community-based tour offers a model for promoting transformative learning and self-reflexivity among medical students while providing a meaningful introduction to the causes and consequences of social-structural inequalities and the complexities of working with vulnerable populations. Our results suggest that such activities may positively influence physicians’ motivations to meaningfully engage with local communities and advocate for underserved populations.
